# Autoantibodies in SLE: Specificities, Isotypes and Receptors

**DOI:** 10.3390/antib5010002

**Published:** 2016-01-04

**Authors:** Barbara Dema, Nicolas Charles

**Affiliations:** Centre de Recherche sur l’Inflammation, INSERM UMR1149, CNRS ERL8252, Université Paris Diderot, Sorbonne Paris Cité, Faculté de Médecine site Bichat, Laboratoire d’Excellence Inflamex, DHU FIRE, Paris 75018, France

**Keywords:** autoantibodies, isotypes, lupus, SLE, Fc receptors

## Abstract

Systemic Lupus Erythematosus (SLE) is characterized by a wide spectrum of auto-antibodies which recognize several cellular components. The production of these self-reactive antibodies fluctuates during the course of the disease and the involvement of different antibody-secreting cell populations are considered highly relevant for the disease pathogenesis. These cells are developed and stimulated through different ways leading to the secretion of a variety of isotypes, affinities and idiotypes. Each of them has a particular mechanism of action binding to a specific antigen and recognized by distinct receptors. The effector responses triggered lead to a chronic tissue inflammation. DsDNA autoantibodies are the most studied as well as the first in being characterized for its pathogenic role in Lupus nephritis. However, others are of growing interest since they have been associated with other organ-specific damage, such as anti-NMDAR antibodies in neuropsychiatric clinical manifestations or anti-β2GP1 antibodies in vascular symptomatology. In this review, we describe the different auto-antibodies reported to be involved in SLE. How autoantibody isotypes and affinity-binding to their antigen might result in different pathogenic responses is also discussed.

## 1. Introduction

Systemic Lupus Erythematosus (SLE) is a chronic autoimmune and inflammatory syndrome whose broad etiology has been described as genetic, epigenetic, hormonal, environmental and immune-regulatory factors to be involved. It affects mostly women at childbearing age and the clinically courses are unpredictable with periods of remission and flares. During the course of the disease many organs may be damaged (such as skin, joints, nervous and vascular systems, or kidneys) leading to a wide clinical heterogeneity. The presence of a large amount of autoantibodies specific to self-antigens mainly of nuclear origin (double-stranded DNA (dsDNA), Smith antigen and ribonucleoproteins (Sm/RNP), anti-Sjögren’s-syndrome-related antigen A and B (SSA/Ro, and SSB/La, respectively) is the hallmark of the disease. The anti-nuclear antibodies (ANAs) are considered markers of diagnosis and prognosis of the disease [1] and significant associations between some autoantibody specificities and clinical features were described [2,3]. However, considering the important panel of new autoantibody specificities reported [4] as well as the diverse mechanisms studied in lupus pathology [5,6] the question whether SLE is a single disease with several phenotypes, or a similar and common phenotype present in a range of different diseases [7], remains debated in the field.

The pathogenesis of the autoantibodies has been the focus of many studies, and, in some cases, the tissue injury and ulterior phenotypic manifestations was shown to develop as a result of autoantibody-mediated mechanisms. This involves accumulation of Immune-Complexes (IC), cell surface binding and cytotoxicity, reactivity with autoantigens expressed on apoptotic or activated cell surfaces, penetration into living cells and binding to cross-reactive extracellular molecules [8,9]. However, the experimental evidence necessary (Witebsky criteria) [10] for some autoantibodies to be pathogenic is far from being elucidated.

The ICs bind Fc receptors, thereby modulating innate and adaptive immune cells responses. The engagement induces the activation of intracellular signaling pathways through inhibitory and/or activating motives (ITIM or ITAM) to inhibit and/or elicit immune functions [11]. Genetic variants of Fc receptors have been associated with SLE susceptibility and SLE severity [12] and the reduced expression on the myeloid cell surface membranes led to the hypothesis of the contribution of a reduced FcR-mediated IC clearance in lupus nephritis [13].

Recently, it has been described that the differences in affinity of antibody and antigen interactions are discriminated by FcR and promote different molecular signals resulting in distinct immunological responses [14]. The presence of autoantibodies in other inflammatory processes (e.g., anti-dsDNA antibodies in bacterial infections [15]), the involvement of low-affinity autoantibodies in autoimmunty [16] as well as the number of self-antigens described in SLE [4], may suggest a different pathological role of autoantibodies in SLE depending on their affinity and their specificity, and may explain the wide immune-mechanisms described, although further studies remain to be done.

Deciphering the specificities and the pathogenic role(s) of autoantibodies in SLE may lead to the development of specific treatments for SLE patients. Recent advances on the use of medications targeting directly or indirectly the B cell compartment (anti-CD20 treatment, Rituximab®; anti-BAFF treatment, Belimumab®, respectively) or normal donors non-pathogenic IgG intravenous infusions (IVIg) correspond to therapeutic evidences demonstrating both the pathogenic role of autoantibodies and the promises raised by their targeting [17,18,19].

This review aims to compile the advances in understanding the functional relevance of the different autoantibody specificities, isotypes and their binding receptors in the pathogenesis of SLE.

## 2. Why Are the Autoantibodies Produced?

SLE is a prototypic autoimmune disease caused by the loss of B cell tolerance and subsequent recognition of self-antigens and becoming autoreactive. During B cell development pre-B cells mature into antibody-secreting plasma cells (PC) and receive multiple positive and negative signals to determine their activation, deletion or anergic status [20,21]. SLE patients and lupus-like disease mouse models reveal many examples of genetic abnormalities that impact different self-tolerance checkpoints leading to overproduction of autoantibodies [22]. Few examples of key findings in the loss of B cell tolerance during SLE pathogenesis are presented below. However, the complex machinery registered in the production of autoantibodies has been extensively reviewed elsewhere [23].

Depending on the survival niche (extrafollicular or germinal center), PCs become short- or long- lived PCs, respectively. This B-cell fate depends on the BCR signal strength signal and the input of signals derived from cells and molecules of the microenvironment. Although central selection reduces auto-reactivity, a great number of immature B cells (40%) are self-reactive and normally found in the peripheral blood of healthy individuals. However, under normal conditions, these autoreactive immature B cells are removed from the repertoire in the bone marrow and during the transition to mature naïve B cells in the periphery [24]. Genetic factors can directly or indirectly influence these selection steps, which in SLE lead to the accumulation of autoreactive mature B cells. B cell endogenous effectors, such as kinases, phosphatases and transcription factors can influence B cell survival, differentiation and selection steps.

As direct or indirect BCR signaling inhibitors, Src-family tyrosine kinase members Lyn, Blk and Csk, play a key role in the selection process occurring during B cell differentiation and maturation. The B-cell hyper-reactivity detected in Lyn-deficient mice is well studied and known to induce an accumulation of autoreactive plasma cells [25,26]. Decreased expression of Lyn in peripheral B cells of SLE patients has been described [27] and a polymorphism in the *Lyn* gene promoter is associated with increased autoantibody titers in a subset of SLE patients [28]. Similarly, a decreased expression of Blk in a lupus-prone environment also induces a general B cell hyperactivity leading to the same consequences as above [29]. Csk increases the inhibitory phosphorylation of Lyn (Y508) which is associated with elevated production of antibodies and transitional B cells [30]. At the same time, a polymorphism in the gene of the tyrosine phosphatase *PTPN22* (C1858T), which is associated with SLE susceptibility, leads to a substitution of a tryptophan to an arginine (R620W) which reduces the binding of PTNP22 to CSK kinase, inducing lower B-cell responses and compromising central and peripheral tolerance [31]. Additionally, some transcription factors determine the balance between B cell and plasma cell identities. Genetic polymorphisms and/or variation in the expression level of these transcription factors have been associated with SLE. For instance, BLIMP-1 (*PRDM1* gene) and XBP1 are transcription factors promoting generation and survival of plasma cells and repressing the B-cell gene expression program factors: ETS1, PAX5 and BCL-6 [21]. ETS1 is reduced in peripheral blood mononuclear cells of SLE patients and *Ets1* ablation leads to an accumulation of PCs, autoantibodies and a lupus-like phenotype in mice [32]. Conversely, deficiency of *PRDM1* reduces the severity of SLE in mice [33].

In addition to these B cell compartment endogenous factors, accumulation of autoreactive plasma cells and autoreactive antibodies can be promoted by exogenous factors directly influencing PC survival and differentiation during SLE biology. Cytokines such as BAFF, IL-4, IL-6 and IFN-α are known to be central in autoantibody production amplification during the disease course. A breakthrough in the study of SLE pathogenesis was the discovery of B-cell activating factor (BAFF), a B-survival factor produced by myeloid cells (monocytes, macrophages, neutrophils and dendritic cells) and activated T cells [34]. Increased levels of BAFF, detected in SLE patients periphery and in lupus-like mouse models, allow autoreactive B cells to be rescued from negative selection, and lead to the formation of germinal centers (GC) and to autoantibody production [35,36]. The efficacy of Belimumab, a monoclonal antibody targeting human BAFF, in the treatment of SLE has been tested in randomized clinical trials, becoming the first approved therapy for a subset of SLE patients [34]. In genetically lupus-predisposed background, high levels of IFN-α (known to be an important player in SLE pathogenesis [37]) induce short-lived plasma cell differentiation and autoantibody production, which is further enhanced by co-stimulatory molecules, Toll-like receptor 9 (TLR9) engagement and BAFF [38]. At the germinal center, T follicular helper cells (T_FH_) are required to induce memory B cell differentiation and long-lived PC to produce class switched and somatically mutated antibodies. Evidences of an aberrant accumulation, over-reactivity and function of T_FH_ cells are described in SLE [39] and lupus-like mouse models. T_FH_ cells influence and are influenced by the overexpression of the cytokine milieu observed in the microenvironment: IFN-γ, IL-17, IL-21 and IL-6 [40]. In addition, basophils are known to support humoral memory responses via their production of IL-4 and IL-6 [41]. These circulating granulocytes are known to accumulate in secondary lymphoid organs in active SLE patients and in lupus-prone *Lyn*-deficient mice where they can support short-lived plasma cell maturation and/or survival [42].

Besides B cell intrinsic abnormalities and influences of the cytokine milieu, autoreactive B cells are supported mainly by the continuous exposure to autoantigens and circulating ICs. Studies in murine models have revealed that naïve autoreactive B cells with low affinity for DNA or RNA associated antigens can be activated through a dual engagement of their surface BCR and intracellular DNA/RNA sensing receptors: TLR7 (ssRNA) and TLR9 (CpG-dsDNA) [20]. Although the molecular mechanisms that mediate this process still need more clarifications, some observations indicate the: (i) the defective clearance of apoptotic bodies [43] and their impaired phagocytosis by macrophages [44]; (ii) neutrophils extracellular traps (NETs) as an additional source of nucleic acids [45]; and (iii) the overexpression of TLR7 (in lupus prone (BXSB) and transgenic mice (Y chromosome autoimmune accelerator, *Yaa* and *Tlr7* duplication)), that drives the proliferation and differentiation of B cells leading to an increased production of antibodies [46,47].

Interestingly, all these mechanisms by which B cells may become pathogenic in lupus-susceptible individuals are completed with a higher complexity level residing in the different specificities of autoantibodies. Indeed, origins and specificities of PCs may determine target organs and their pathogenic role (see below). The different origin of antibody secreting cells is consistent with the finding that some serum titers of an autoantibody with a given specificity fluctuate with disease activity (anti-dsDNA) whereas others are not altered (anti-Sm, anti-Ro, anti-La, anti-cardiolipin) [38]. Recently, it was reported that during SLE flares some of the circulating antibody secreting cells were highly polyclonal and they did not recognize the most prevalent lupus antigens, consistent with their differentiation outside of germinal centers. Additionally, a distinct subset was derived from activated naïve B cells, with lower clonality (germline encoded autoantibodies) that persisted in the circulation for several months [48].

If the loss of tolerance by the B cell compartment described above is leading to the appearance of autoantibodies in SLE individuals, some of their various specificities are very well described and are associated with several disease parameters.

## 3. Autoantibody Specificities: An Antigenic Detonation

In contrast to other autoimmune diseases, SLE shows a broad spectrum of self-antigens. Many of them are still unknown and others lack determination and understanding of their pathogenicity. However, we divide self-antigens into two categories: common and newly described antigens, based on: (i) their validated role in SLE pathogenesis and their inclusion in diagnostic criteria from the ACR (American College of Rheumatology) and from the SLICC (the Systemic Lupus International Collaborating Clinics) [49,50] or (ii) their recent discovery and their yet not described pathogenic role.

### 3.1. Common Antigens

The biology of the autoantibodies is crucial for a better diagnosis and prognosis of the disease. The detection of some autoantibodies is part of the clinical diagnostic criteria [49] (ANAs, anti-dsDNA, anti-Sm, anti-phospholipid) either because of the high specificity of their presence in SLE patients or because of their demonstrated pathogenicity. However, apart from autoantibodies, other factors like the clinical symptomatology are considered for diagnosis since the immunological parameters may vary depending on targeted organ and disease activity at the time of assessment, therefore reducing their sensitivity. A close monitoring of the patients is then necessary for a correct diagnosis.

Arbuckle *et al.* [51] described three distinct phases concerning the levels of autoantibodies during disease development. During the first phase, called normal immunity, patients are asymptomatic and they do not show any detectable autoantibody levels. During the second phase, called benign autoimmunity, patients develop positive immunological parameters (ANAs, anti-Ro, anti-La and antiphospholipid antibodies) but lack immediate clinical manifestations. The third phase, corresponding to pathogenic autoimmunity, is defined by the presence of anti-dsDNA, anti-Sm and anti-RNP and is quickly followed by the onset of clinical symptomatology of the disease [51]. Therefore, the presence or absence of autoantibodies as well as their specificity are useful in clinics for diagnosis and prognosis purposes.

Moreover, based on their specificities, some autoantibodies can define a specific outcome of the disease and four different clusters have been proposed: (i) the dsDNA cluster which is associated with a high incidence of renal involvement and high risk of renal damage; (ii) the Sm/RNP cluster which is associated with higher incidence of pulmonary arterial hypertension and Raynaud’s phenomenon; (iii) the anti-cardiolipin and lupus anticoagulant cluster which is associated with neuropsychiatric involvement, antiphospholipid syndrome and hemolytic anemia; and (iv) the Ro/La cluster which does not show any clinical association [52]. However, as described below, many other autoantibodies might play an important role in these and other clinical manifestations. Some features of common autoantibodies (prevalence, targets, isotypes and demonstrated pathogenic role) are summarized in Table 1.

**Table 1:** Common self-reactive antibodies and their isotype-differential pathogenic mechanisms described in Systemic Lupus Erythematosus (SLE).

Autoantibody specificity	Prevalence (%)	Ref.	Isotype	Diagnostic marker (SLICC)	Association with disease activity	Pathogenesis involvement	Ref.
**ANA**	95	[7]	IgG	yes	no	Autoimmune disease	[53]
	34	[54]	IgA	no	no	cutaneous lupus	[55]
**dsDNA**	60–90	[56]	IgG	yes	yes	nephritis, skin and cerebral lupus	[57,58,59,60]
	63	[61]	IgG1	no	yes	LN	[62]
	4	[61]	IgG2	no	no	-	-
	14	[61]	IgG3	no	no	complement activation, LN	[63]
	7	[61]	IgG4	no	no	-	-
	52	[64]	IgM	no	yes	negative association with LN	[64,65]
	40	[5]	IgE	no	yes	LN	[66]
	49	[67]	IgA	no	yes	LN	[67]
**Nucleosome**	50–90	[68]	IgG	no	yes	LN	[68]
	15	[69]	IgG3	no	yes	LN	[70]
**Sm**	20–40	[71]	IgG	yes	no	renal, neurologic, vasculitis and hematologic disorders	[71]
	58	[61]	IgG1	no	no	-	-
	40	[61]	IgG3	no	no	-	-
	40	UP	IgE	no	no	-	-
**snRNP**	20–30	[72]	IgG	no	no	-	-
	65	[73]	IgM	no	no	-	-
**SSA/Ro**	30–40	[74]	IgG	no	no	neonatal lupus	[75]
	30	UP	IgE	no	no	-	-
**SSB/La**	10–15	[76]	IgG	no	no	neonatal lupus	[75]
	25	UP	IgE	no	no	-	-
**PL**	30–40	[77]	IgG	yes	no	hematologic involvement	[78]
**β2GP1**	10–35	[79]	IgA	no	no	thromboembolic events	[79]
**C1q**	20–50	[80]	IgG	no	yes	LN	[81]
**Rib P protein**	10-40	[82]	IgG	no	no	neuropsychiatric symptoms, liver disease	[82]
**NMDAR**	30	[83]	IgG	no	no	neuropsychiatric symptoms	[84]

Abbreviations used: ANA: anti-nuclear antibodies; dsDNA: double stranded DNA; Sm: Smith; snRNP: small nuclear ribonucleoprotein; SSA: Sjögren Syndrome antigen A; SSB: Sjögren Syndrome antigen B; PL: phospholipids; 2GP1: 2-glycoprotein 1; Rib: ribosomal; NMDAR: N-Methyl-D-Aspartate Receptor; LN: Lupus Nephritis; UP: Unpublished.

#### 3.1.1. Anti-Nuclear Antibodies (ANAs)

For more than 60 years the detection of ANAs by indirect immune-fluorescence (IIF) staining has been fundamental for the diagnosis of autoimmune diseases. The prevalence of ANAs among SLE is very high, but they may also be detected in patients with other autoimmune, malignant or infectious diseases as well as healthy controls [53].

The IFF technique is performed on the HEp-2 cell line and it was first described by Coons and Kaplan [85]. Currently, it is used to diagnose and to follow-up many autoimmune diseases, but the results obtained are subjected to several recommendations that were summarized by Agmon-Levin *et al.* [53].

The term ANA is not considered accurate because it embraces antibodies directed against components localized in several cellular compartments including nuclear constituents, nuclear membrane, mitotic spindle apparatus, cytosol, cytoplasmic organelles and cell membranes. The analysis of the immunofluorescence staining shows different patterns that reflects the topographic distribution of target autoantigens and may suggest significant information about antibody specificities. For example, a homogeneous nuclear pattern may be related to the detection of antibodies against dsDNA, histones and chromatin/nucleosomes, whereas a coarse speckled nuclear pattern may correspond to Sm detection. Thus, the interpretation of the results depends on clinical circumstances and additional antibody specificity testing [53].

#### 3.1.2. Anti-dsDNA Antibodies

Anti-dsDNA antibodies are considered a diagnostic marker and one of the classification criteria for SLE [49]. They were first described in sera of SLE patients in 1957 [86,87] and the actual prevalence described is 60 to 90% of patients. However, the debate of an additional and more specific description of the specificity of anti-dsDNA antibodies as a diagnostic criteria is currently opened [56]. Compagno *et al.* reported that the detection method used in clinical practice determines its specificity for SLE diagnosis and an increased positive predictive value is reached when multiple assessments are done in parallel in several clinical centers [88]. Moreover, the anti-dsDNA positivity using different techniques not only results in a variation of associations with clinical and biochemical manifestations of SLE but with other rheumatic and inflammatory conditions, suggesting the urge of a consensus detection method among centers as well as the consideration of a positive anti-dsDNA test result as an only complimentary diagnostic tool [89].

*In vivo*, the DNA that is introduced to the immune system is part of a chromatin structure containing histones (in octamers called nucleosomes) and DNA being its major constituent [90]. But antibodies against mammalian dsDNA, detected by different laboratory techniques in SLE sera, are specific for different structures: the elongated nucleosome linker B-DNA (double-helix, right handed turn), phosphodeoxiribose backbones; the higher-order bent DNA structures such as those in the nucleosome or in *Crithidia luciliae* kinetoplast DNA; the ssDNA regions that may be present within (nucleosomal) dsDNA; the Z-DNA and cruciform DNA structures (left handed turn) [91]. However, just the Z-DNA structure is demonstrated to be immunogenic by itself and is able to induce antibodies in a hapten-carrier context (hapten as a non-immunogenic molecule that elicits an inflammatory response when bound to a carrier protein) [92].

Several hypotheses have been described to explain the immunogenic properties of the dsDNA and the origin of anti-dsDNA antibodies. One of these theories is the presence of cross-reacting antigens such as phospholipids [93], α-actinin [94], laminin [95], entactin [96], the decapeptide DWEYSVWLSN [97], among others [57]. But this theory is difficult to maintain unless the cross-reacting antigen has structural and charge similarities to DNA. Indeed, the primary response to DNA might be oligospecific, but the secondary is predicted to affinity-mature towards the non-DNA immunogenic rather than towards DNA and therefore the specificity to DNA should die out [15]. To elicit a persistent immune response, a T helper (T_H_) cell stimulus is necessary for dsDNA-specific B cells. Different nucleosome proteins (high affinity peptides to chromatin) have been suggested to drive this process by being presented in the context of MHC class II molecules and the appropriate costimulatory molecules to T_H_ cells [98]. Therefore the presence of anti-dsDNA antibodies seems to predict the presence of a spectrum of other antibodies that are specific for chromatin ligands and structures. This hypothesis was based on the studies done with the peptide derived from *Trypanosoma cruzii* (Fus1) [99] and the T cell antigen from polyomavirus BK [99] that bind DNA and are highly immunogenic. In this case, chromatin (dsDNA, histones, or tertiary or complex structures) represents the hapten for a repertoire of B cells, whereas the viral/parasitic DNA-binding proteins represent the carrier protein. The T cell response is directed against the carrier protein, but a humoral immune response is elicited against chromatin. This corresponds to the hypothesis of an infectious origin. However in SLE, a histone molecule of autologous origin might represent the carrier protein, activating non-tolerant T cells. Although the B cell repertoire response is limited to the exposed determinants on the chromatin surface, just a few peptides may be sufficient to activate T_H_ cells with the potential to stimulate the whole array of chromatin-specific B cells, explaining the comprehensive repertoire of chromatin-reactive IgG antibodies in SLE patients [15]. The infectious origin model might describe the production of anti-dsDNA antibodies transiently as the carrier protein is expressed temporarily. By contrast, in the autologous origin there is a sustained production of anti-dsDNA autoantibodies, which is limited by the toleration of autoimmune T cells. These different processes would probably not have the same impact on SLE, because this transient versus sustained stimulation might lead to the production of low antibody levels with low avidity, or high antibody levels with high avidity, respectively [15]. However, both models may be complementary since flares of the disease are often associated with a benign viral infection [100]. Given that viral infections may initiate a disease flare in SLE patients, that an initial presentation of SLE mimics infections and that both kinds of carriers share the same haptens, the heterologous transient model may lead to the establishment of a more autologous sustained stimulation.

Considering cross-reacting ligands, complement activating antibodies, and Fcγ-receptor binding subclasses as properties that confer pathogenicity, not all anti-dsDNA antibodies are pathogenic (Witebsky’s criteria [10]). However, Rekvig propose the model of chromatin exposure to be considered as a pathogenic evidence, useful for a more accurate diagnosis of SLE [56].

As mentioned above, anti-dsDNA antibodies are associated with lupus nephritis (LN) and renal manifestation of the disease. The pathogenic mechanism is described in two ways. First, the chromatin fragments are exposed at the glomerular basement membrane (GBM) (chromatin model), and some anti-dsDNA antibodies can cross-react with GBM components such as α-actinin [94], laminin [95], or entactin [96]. The chromatin fragments are associated with high affinity to membranes and matrices and thus targeted by autoantibodies *in situ* [101]. Second, a preformation of ICs in the circulation between chromatin fragments and anti-dsDNA antibodies allows their deposition and binding to the glomerulus afterwards. The early phase of mesangial nephritis might be initiated by circulating IC, but progressive nephritis is correlated with the loss of renal DNase I, the accumulation of large chromatin fragments in the GBM and the *in situ* formation of ICs in lupus-like mouse models [58,102]. Moreover, the chromatin fragments were shown to possess themselves high affinity for renal laminin and collagen [101]. Therefore the chromatin of dying cells is retained in the glomerulus, amplifying *in situ* IC formation. This data suggest that anti-dsDNA antibodies do not have an a priori nephritogenic effect in the absence of chromatin but they are nephritic when chromatin is exposed at the glomerulus [15] as determined with TEM (transmission electron microscopy) [103] and immune electron microscopy (IEM) [104].

Besides LN, similar processes have been observed for other lupus-driven organ damages. For instance, electro-dense structures in skin contain chromatin-IgG complexes which show a high affinity for dermo-epidermal laminin and collagen [59,60]. This may lead to the same pathogenic mechanism as the one described above for the kidney. Another example resides in a subset of dsDNA antibodies which have been found to cross-react with a consensus amino acid sequence (D/EWD/EYS/G) present in the NR2A and NR2B subunits of the NMDA (N-methyl-D-aspartate) glutamate receptor. Thus, these anti-dsDNA antibodies may induce neuronal death via an apoptotic pathway and mediate non-thrombotic and non-vascular abnormalities of the central nervous system [57].

Anti- mitochondrial DNA antibodies were recently found to have a better correlation with LN than anti-dsDNA antibodies. Mitochondrial DNA has been detected along with NETs in LN renal biopsies and additionally mitochondrial DNA/anti-mitochondrial DNA antibody complexes were described to induce plasmacytoid dendritic cells IFNα production (important in the pathogenesis of the disease) greater than dsDNA/anti-dsDNA antibody complexes [105,106].

#### 3.1.3. Anti-Nucleosome Antibodies

Nucleosomes, as briefly explained earlier, are complex structures in which histone octamers are surrounded by chromatin [90]. This primary structure is considered as the core nucleosome particle. However, nucleosomes *in vivo* are plastic and dynamic structures which are associated with several other particles such as RNA, ribonucleoproteins, transcription factors and enzymes [107].

Although anti-nucleosome antibodies may be also found in other autoimmune diseases such as systemic sclerosis, the prevalence of these antibodies in sera of SLE patients is higher and is considered a more sensitive marker compared to dsDNA antibodies [68,70,108]. Still, the diagnostic use of this parameter has been limited because the enzyme-linked immunosorbent assays (ELISA) used to determine the presence of anti-nucleosome antibodies also detect a broad variety of antibodies such as histones, dsDNA and other structural determinants, making it difficult to correlate the results with disease processes. Recently, newer immune assay platforms for detecting anti-nucleosome and anti-dsDNA antibodies have shown promising results in assessing disease activity, particularly when anti-dsDNA antibodies are negative [109].

As mentioned earlier, nucleosomes may play an important role in SLE through the induction of a T cell mediated response by the hapten carrier-like system to raise several autoantibodies [15]. Additionally, the understanding of the origin of those nucleosomes that drive the immune response is becoming more complex. Apoptotic bodies may be generated by an aberrant apoptosis coupled to a reduced clearance by phagocytes [110], by necrotic or damaged cells, by circulating microparticles [111] and by NETosis corresponding to extracellular traps extruded from neutrophils (NETs) [112].

HMGB1 (high mobility group box-1) is a non-histone DNA-binding protein found to be released to the extracellular space in the chromatin-particles derived from apoptosis, necrosis or NETosis. It acts as a damage-associated molecular pattern (DAMP) or alarmin inducing either cytokine release by monocytes, cell migration, wound healing and neovascularization [113]. In the last few years, the discovery of HMGB1 as an autoantibody target has been of particular interest due to its proinflammatory role in many autoimmune and inflammatory processes [113]. Moreover, HMGB1 acts as well as an autoadjuvant in the induction of immune responses in SLE [114]. HMGB1-containing ICs activate plasmacytoid dendritic cells to produce IFN-α, an important cytokine in lupus pathogenesis, as described above [113]. In addition, HMGB1-containing nucleosomes from apoptotic cells induced anti-dsDNA and anti-histone IgG responses in a Toll-like receptor (TLR) 2-dependent manner [114].

Anti-nucleosome antibodies have been associated predominantly with severity of renal involvement in human SLE [115,116] and are highly correlated with disease activity [68,116] and anti-dsDNA antibody titers [117]. In murine lupus models, anti-nucleosome antibodies precede many other autoantibodies. Moreover, nucleosome specific CD4+ T cells are detected earlier than the pathogenic autoantibodies, suggesting that they might play a role in the onset of the disease [118]. However, in another lupus-like mouse model, anti-nucleosome antibody generation *in vivo* is described as depending on the expression of TLR9 unlike the production of anti-dsDNA antibodies. Interestingly, only the latter kind of autoantibodies drives the development of lupus-like proliferative nephritis [119].

#### 3.1.4. Anti-Sm Antibodies

Sm antigens (named after their identification in the serum from a patient named Stephanie Smith) are a set of seven core proteins (B, D1, D2, D3, E, F, G) forming a ring for small nuclear ribonucleoproteins (snRNP). SnRNPs sharing the ring core structure of Sm antigens are U1, U2, U4 and U5, and are essential cofactors for pre-mRNA splicing and assist in the removal of introns from pre-mRNA. The Sm core proteins are assembled in the cytoplasm where they bind to the snRNP before they are transported to the nucleus [120].

The anti-Sm antibodies are directed against several epitopes distributed all over the core proteins, the B protein being the major antigen followed by D1 and D2 [121]. Talken *et al*. described an epitope that induces a T cell immune response localized in the region between the two highly conserved and shared sequence motifs among the core proteins, designated as Sm1 and Sm2. This region is inside the core particle where the interaction with the snRNPs occurs [122]. Moreover, the proline rich sequence PPPGMRPP, repeated three times on the C-terminus of the B protein of the Sm core, was found to be similar to the sequence (PPPGRRP) in the Epstein-Barr nuclear antigen 1 (EBNA-1) of the Epstein Barr virus (EBV). That observation has led to the hypothesis that an infection with EBV in predisposed individuals may induce lupus by cross-reacting epitopes [123].

The pathogenic role and the contribution of anti-Sm antibodies to the disease remain uncertain. However, they are highly specific for SLE and represent one of the immunological diagnostic criteria for the disease [49]. Nevertheless, the sensitivity is low and they are only detected in 20% of Caucasian SLE patients and 30%–40% of African, African-American and Asian patients [71]. Supported by the isolation of anti-Sm antibody from ICs in the glomeruli of LN patients [124], their contribution to renal failure has been suggested. However, many contradictory associations reported in several studies [71] prevent anti-Sm antibodies being a good biomarker of LN and additional studies are needed to confirm this association.

Recently the possible neural toxicity of anti-Sm antibodies has been described [125], adding another player (with anti-NMDAR antibodies, see below) to the pathogenesis of the neuropsychiatric manifestation of SLE (NPSLE). Anti-Sm antibodies are found to react with neuroblastoma cell lines and they are also detected in the cerebrospinal fluid of NPSLE patients and their levels are correlated with anti-NR2 antibodies.

#### 3.1.5. Anti-RNP Antibodies

The snRNP are RNA-protein complexes, abundant in the nucleus and involved in the nuclear processing of the pre-mRNA along with other proteins constituting the spliceosome. The anti-RNP antibodies react with proteins (70 kDa, A, C) that are associated with the U1 RNA forming the U1snRNP [126]. The 70 kDa protein is one of the major determinants in the antibody response to U1-RNP: anti-70 kDa antibodies are developed early in SLE pathogenesis and may contribute to the development of antibodies against other proteins of the U1-RNP complex through the epitope spreading mechanism [127]. Cellular oxidation and apoptosis contribute to this process since they lead to a modification of autoantigens able to increase or decrease antibody affinity [128,129].

Anti-U1-RNP antibodies are detected in 20%–30% of SLE patients but they do not show a good specificity for SLE since they are commonly found in mixed connective tissue disease (MCTD) [130]. However, the isotype of the antibody response may show the pathogenic mechanism or the role of these autoantibodies in the disease since IgM anti U1-RNP antibodies where predominantly found in SLE whereas IgG anti-U1-RNP in the absence of IgM anti-U1-RNP antibodies are found in MCTD [73].

Anti-RNP antibodies have been associated to Raynaud’s phenomenon [126], although limited studies to understand the mechanism were reported.

#### 3.1.6. Anti Ro/SSA and anti La/SSB Antibodies

Ro/SSA and La/SSB are ribonucleoproteins (RNPs) included in the complexes uridine rich hY (human cytoplasmic) RNAs. Ro/SSA comprises four different proteins (45, 52, 54, 60 KDa), Ro52 and Ro60 being the more deeply studied. La/SSB is a phosphoprotein of 48 KDa that binds to a variety of small RNA, including 5S cellular RNA, tRNA, 7S RNA, all of which are transcribed by RNA polymerase III. These proteins can be found in the nucleus and are transported to the cytoplasm after assembly of the Ro/La RNP complex. Under certain circumstances such as stress, UV radiation, apoptosis or viral infection, these proteins are found on the cell surface [131].

Several epitopes are recognized by the anti-Ro and anti-La antibodies [131]: linear epitopes, consisting on the sequence of amino-acids; cryptic epitopes, linear epitopes hidden within the native structure of the antigen (Ro60 epitope is cryptic, masked by the binding to hY RNA) and apotopes, epitopes uniquely expressed on the surface of apoptotic cells. Anti-Ro and anti-La antibodies are found in 30%–40% and 10%–15% of SLE patients, respectively, but are more frequent in Sjögren syndrome patients constituting a marker for its diagnosis [76]. No pathogenic mechanism has been attributed to anti-Ro and anti-La antibodies in SLE disease. However, women with positive levels to these autoantibodies show a high risk to develop neonatal lupus in the fetus with congenital heart block as the most serious symptom of the syndrome [75]. Anti-La antibodies have been found to cross the placenta and form IgG-apoptotic complexes in the fetal heart in a passive transfer model. In addition, *in vitro* opsonization of apoptotic cardiocytes by anti-Ro and anti-La IgGs leads to the inhibition of the apoptotic clearance. These observations suggest a model where anti-Ro/La antibodies bind to apoptotic fetal heart cells during embryonic remodeling, thereby preventing their clearance by infiltrating macrophages and causing tissue injury [132].

An interesting phenomenon described is the epitope spreading detected after the first immunization with Ro60 autoantigen. Indeed, B and T cell immune response diversify at the level of specificity, from a single determinant of a protein to many different sites of the same protein, or other different proteins. Thus, a single immunization of Ro60 was shown to lead to the production of antibodies to La and other spliceosome proteins [133]. This may recreate the model of the humoral response in SLE (see above).

#### 3.1.7. Anti-Phospholipid Antibodies

Anti-phospholipid antibodies (aPLs) are found in 30%–40% of SLE patients but they are not specific and can be detected in other autoimmune diseases, infections and drug induced disorders, as well as in some healthy controls [134]. Around half of SLE patients with aPLs develop antiphospholipid syndrome, an autoimmune disorder characterized by recurrent arterial or venous thrombosis, pregnancy-related problems, thrombocytopenia, hemolytic anemia and persistent elevated levels of aPLs [134].

APLs comprises a group of antibodies that recognize anionic charged phospholipids including cardiolipin, phosphatidylserine, phosphatidylinositol, phosphatidylglycerol and phosphatidic acid; neutrally charged phospholipids including phosphatidylethanolamine, phosphatidylcholine, platelet activating factor (PAF), sphingomyelin and a phospholipid binding protein cofactor called β2-glycoprotein 1 (β2GP1) [130]. The laboratory test to determine aPL positivity include the detection by ELISA of anticardiolipin and β2GP1 antibodies by ELISA and functional lupus anticoagulant assay that demonstrates the ability of aPLs to prolong phospholipid-depending clotting reactions. The latter test has been shown better correlation with clinical symptoms than the ELISA [77].

The presence of aPLs is necessary but not sufficient for pathological thrombus formation. They create a prothrombotic state that increases the risk of thrombosis but the presence of an additional prothrombotic factor is required. Several proposed mechanisms has been described in the thrombus formation: disruption of procoagulant and anticoagulant reactions on cell membranes through aPLs’ binding to proteins implicated in clotting regulation (prothrombin, factor X, protein C and plasmin); interaction of aPLs with specific cell-surface receptors (proteins, lipids or both) and induction of signaling transduction resulting in an up-regulation of cell-surface proteins; conformational and oxidative post-translational modifications of β2GP1, leading to a reduction of the activity of endothelial nitric oxide synthase and complement activation [135].

Many thrombotic events in the kidney have been associated with aPLs such as renal artery stenosis, renal infarction, renal vein thrombosis and thrombotic microangiopathy (TMA); all of them leading to a phenomenon described as aPL-associated nephropathy. This nephropathy is affecting 10.4%–34% of SLE patients [78].

Studies of anti-galectin antibodies as a novel biomarker for the development of a secondary antiphospholipid syndrome in SLE patients have been revolutionary. Galectins are glycan-binding proteins and they mainly regulate extracellular processes including cell-cell and cell-matrix interactions, apoptosis and cytokine secretion [136]. Elevated levels of anti-galectin-1, 2, 4, 7, 8, 9 antibodies have been found in patients with SLE, anti-galectin-8 being the most prevalent one, reaching almost 40%. Anti-galectin-2 is highly correlated with Lupus anticoagulant and anti-cardiolipin antibodies, suggesting their importance in antiphospholipid syndrome diagnosis in SLE [137].

#### 3.1.8. Anti-C1q Antibodies

Complement component C1q is the first molecule in the classical pathway of complement, and the key for the activation of the entire cascade. Although, the classical pathway is known to be initiated in an antibody-dependent way (IgM or IgG complexes), C1q molecules can activate complement by recognizing different structures directly on microbial or apoptotic bodies, or through endogenous pattern recognition molecules (PRM) such as immunoglobulins and pentraxins (C-reactive protein, CRP) [138].

C1q deficiency has been described as a high risk factor to develop SLE [139] but the genetically C1q deficiency in SLE is very rare [140] and the susceptibility risk of gene variants at the *C1Q* gene remains controversial [141,142]. Conversely, low levels of C1q are typically associated with disease flares and with the appearance of anti-C1q antibodies [80] which, in turn, are found in 20%–50% of SLE patients and in up to 100% of active proliferative LN patients [81].

Anti-C1q antibodies are directed against a cryptic-epitope on the collagen-like region (CLR) exposed on bound C1q [143]. Specifically, they target C1q bound on early apoptotic cells but not C1q conjugated to immunoglobulin (Igs), to ICs or even to late apoptotic/necrotic cells [144]. It is described that purified anti-C1q antibodies from LN patients can inhibit phagocytosis of early apoptotic cells, contributing to the accumulation of apoptotic bodies [145].

Although the levels of anti-C1q antibodies and hypocomplementemia are correlated in SLE [80], there is limited evidence of the pathological mechanisms involved. Recently, it was demonstrated that SLE patient-derived anti-C1q can activate the complement system *in vitro*. Anti-C1q IgGs predominantly activate the classical pathway; anti-C1q IgAs activate the lectin pathway, whereas anti-C1q IgMs may trigger both pathways [146]. Considering the deposits of Igs (IgG, IgM, IgA) and complement proteins (C1q, C3, C4, Mannose Binding Lectin (MBL)) detected in SLE patient kidney biopsies [147,148] as well as the quantity of anti-C1q deposited in these glomeruli [149], a contribution of the complement activation in the kidney to the development of LN was suggested [148].

#### 3.1.9. Anti-Ribosomal P (anti-P) Antibodies

Anti-P antibodies recognize three ribosomal proteins of the 60S subunit designated as Rib-P0, Rib-P1 and Rib-P2, with molecular weights 38, 19 and 17 kDa, respectively [150]. Ribosomal P proteins are localized in the cytoplasm of cells where they control the gene expression process. However, Rib-P0 can be found on the surface of some cells including human hepatoma cells, neuroblastoma cells, fibroblast, endothelial cells, glomerular mesangial cells, human monocytes and T-cells. The surface exposure is induced by cellular activation or apoptosis [82,151]. Human peripheral blood monocytes show ribosomal-P proteins on their surface after activation and more abundantly in annexin V positive ones (apoptotic). They bind highly purified anti-P antibodies, inducing the secretion of the proinflammatory cytokines IL-6 and TNF-α, independently of FcγR cross-linking [152]. Later, the same group demonstrated that anti-P antibodies might contribute to the TH1 response by inducing the secretion of IL-12 and IFN-γ by activated monocytes [153].

Although the presence of anti-P antibodies is highly specific for SLE, the prevalence is low and variable (10%–20% to 40%) and depends on the detection assay used [154]. The ELISA based on the combination of all three recombinant proteins resembling the native hetero-complex as antigen seems to be more accurate as a diagnostic marker than the total native ribosomal complex or the recombinant carboxyl-terminal amino-acid sequence shared by three ribosomal P proteins [154]. Several groups have published the association of anti-ribosomal P antibodies with some clinical features of SLE: neuropsychiatric lupus, LN and lupus hepatitis [82] but the pathological mechanism attributed to anti-P antibodies remains uncertain.

The most frequent association described has been between the SLE neuropsychiatric symptoms (psychosis, depression) and anti-P antibodies levels [82], but their utility as a diagnostic marker remains controversial due to the negative results obtained in an international meta-analysis [155]. However, recent studies described that mice transferred passively with anti-P antibodies showed smell alterations, depression-like manifestations and memory impairment. The anti-P antibodies cross-react with a plasma membrane protein called neuronal surface P antigen (NSPA), which exposes a P epitope, and is expressed at specific brain regions including areas involved in memory, cognition and emotion [156]. NSPA is involved in glutamatergic synaptic transmission and plasticity related to memory and anti-P antibodies mediate a deleterious effect in these processes [157]. Studies done in HepG2 cells (liver hepatocellular carcinoma cells) show an increase of internalized anti-P antibodies (binding and penetration independently of Fcγ receptors) affecting synthesis of apolipoprotein B and resulting in cholesterol and lipid droplet accumulation. This causes cellular dysfunction which may be related to the liver disease occurring in some lupus patients [158].

Anti-idiotypic antibodies are found in some healthy controls and SLE patients. Some of these anti-idiotypic IgG antibodies are specifically raised against anti-P antibodies idiotype and are highly cross-reactive. They can prevent the binding of anti-P antibodies to their antigen [159]. This might suggest that anti-P positive SLE patients lack anti-idiotypic antibodies, leading to their pathogenic role, but further studies are necessary to confirm this hypothesis.

#### 3.1.10. Anti-NMDAR Antibodies

Anti-NMDA glutamate receptors antibodies (as mentioned before) were described by DeGiorgio *et al*. as a group of anti-dsDNA antibodies that cross-react with a consensus peptide sequence (D/EWE/DYS/G) present in the NR2A and NR2B subunits of the NMDA receptor [57]. Moreover, mice immunized with this consensus sequence produce both anti-DNA and anti-NMDAR antibodies [57,97]. They do not exhibit neuronal damage unless there is a rupture in the blood-brain barrier induced either by bacterial LPS (leading to hippocampal neurons death and memory deficit) [160] or by epinephrine (leading to basolateral amygdale neurons death and disturbances in fear-conditioning) [161]. The anti-NMDAR antibodies bind the open receptor pore only in neurons with activated synapses. They increase the open-state duration of the pore, a function always regulated by glutamate. Higher levels of the autoantibodies are necessary to induce neuronal stress as compared to the levels inducing electrophysiological changes in NMDAR-mediated synaptic transmission [162].

The prevalence of anti-NMDAR antibodies is approximately 30% in SLE patients [83] and their levels are highly correlated with neuropsychiatric clinical manifestations [84]. Moreover, anti-NMDAR antibodies were found in lupus brain and in the cerebrospinal fluid, implying that the break of the blood-brain barrier occurs in SLE even without a history of inflammatory events at the central nervous system [163]. This current data suggest the possible diagnostic marker of anti-NMDAR antibodies in neuropsychiatric manifestations of SLE, mostly when they are detected in cerebrospinal fluid.

### 3.2. Newly Described Antigens

In the last few years the advances in proteomics and epigenomics have allowed the detection of a great variability of novel autoantigens providing new insights into SLE pathogenesis as well as different and promising biomarkers for the diagnosis of SLE.

As mentioned above, some post-translational modifications of self-antigens generate new antigens to which the immune tolerance does not apply. The pathogenic process in SLE creates an inflammatory milieu that promotes the generation of these post-translational modifications. This kind of modifications can be induced by several events, like apoptosis or NETosis. However, each event may induce different modifications and the combination of these modifications might induce autoreactivity [164].

Interestingly, the immune responses from T and B cells against these modifications are different. T cell responses tend to be strictly specific to the modified antigen rather than the native form of the protein, whereas B cell responses tend to be more promiscuous due to the ability of antibodies to bind flanking amino-acid sequences in the modified and native proteins. Mice immunized with the isoasparyl-modified form (isoAsp) of snRNP D produce T cells that only proliferate in response to the isoAsp snRNP D peptide. However, they generate antibodies that bind the isoAsp and Asp form of the peptide but also other lupus autoantigens [165]. Thus, the post-translational modifications may lead to epitope-spreading, a phenomenon widely discussed above and in the field of autoimmunity [166].

Histones are found to be susceptible to post-translational modifications, such as isomerization, acetylation, methylation and citrullination. These histone modifications are increased during both apoptosis and NETosis processes [164]. Histones are DNA-binding nuclear proteins (H2A, H2B, H3 and H4) assembled in an octameric complex, being part of the already discussed nucleosome [90]. The prevalence of anti-histone antibodies in SLE ranges from 17% to 95%, but is higher (67% to 100%) in drug-induced lupus (DIL) [167]. However, these autoantibodies are not SLE specific and can be found in other rheumatic diseases (including Rheumatoid Arthritis, Vasculitis, MCTD, and Sjögren Syndrome). The correlation of anti-histone antibodies with SLE clinical features has been inconsistent, probably due to a higher reactivity to higher-ordered structure nucleosomes (Histone-chromatin complex) than to complexed histones themselves. However, modified histones show a higher immunogenicity [168]. Specific patterns of hyperacetylations on histones (H2A, H2B and H4) as well as specific methylation patterns on H3 have been associated to apoptosis, NEtosis and SLE [169,170].

Molecular mimicry is found to be another phenomenon contributing to the SLE pathogenesis. It is based on the presentation of epitopes from infectious agents, structurally similar to self-antigens, which will confuse the immune system, generating an autoimmune response. Thus, autoimmunity might be primarily triggered through cumulative immune responses of repeated infections during childhood, follow by a boost occurs in predisposed individuals when a specific pathogen load or a unique combination of pathogens is reached [171]. The best known example of this molecular mimicry is the similarity between the octapeptide, PPPGMRPP of the Sm autoantigen and the PPPGRRP from the protein EBNA-1 of the EBV, which also binds to anti-Sm autoantibodies [123]. In addition, cross-reactivity has been also described between the first epitope of the Ro60 peptide (TKYKQRHGWSHKD) and EBNA-1 (GGSGSGPRHDGVRR), which also binds to anti-Ro antibodies [172]. Similarly, other viruses may be involved in SLE pathogenesis. Parvovirus B19 has been associated with SLE and might be responsible for the production of autoantibodies directed against anti-single stranded DNA (ssDNA), that are detected in 30%–70% of SLE patients [173]. HRES-1 human endogenous retrovirus encodes a 28 kDa nuclear protein which cross-reacts with the 70 kDa protein of the U1 snRNP. Moreover, anti-HRES-1 antibodies have been associated with SLE predisposition [174].

Cross-reactivity has been studied as well with regards to anti-dsDNA antibodies, to understand their involvement in the disease. Some anti-dsDNA antibodies are considered nephritogenic either by their interactions with exposed chromatin or by cross-reactive interaction with glomerular antigens expressed on the surface of podocytes and mesangial cells (annexin A2 [175], laminin [95]) or glomerular basement membrane [103]. However, anti-dsDNA antibodies represent only 10 to 20% of the IgG deposition eluted from LN patient kidneys [124]. Thus, seeking other nephritogenic autoantibodies which do not cross-react with dsDNA antibodies has been the aim of many studies. The identification of anti-α-enolase antibodies eluted from kidneys (50% of LN biopsies) of active nephritis patients has been a potential candidate of LN biomarker [176]. Anti-α-enolase antibodies can be detected in 27% of SLE serum samples (70% of active lupus nephritis) but have a poor specificity since they are also reported in mixed cryoglobulinemia (MC), systemic sclerosis (SSc), vasculitis and other rheumatic diseases [177]. Recently, Bruschi *et al*. described that the association of anti-α-enolase antibodies with active LN (serum and kidney biopsies) is isotype- (IgG2) and epitope-dependent, suggesting that different parts of the protein become immunogenic under different conditions. Thus the presence of IgG2 antibodies against α-enolase (together with annexin AI) was shown to be considered as a biomarker of LN allowing its differentiation from other glomerulonephritis [176]. The cytoplasmic enzyme α-enolase has been described in numerous tissues and its expression on the surface of the cell membrane of glomerular and endothelial cells may be induced by inflammatory stimulus [178]. During LN, α-enolase is overexpressed in the kidney and is present in active inflammatory lesions, but the mechanisms underlying this process is not clear yet [179].

Anti-annexin antibodies have been also detected in LN biopsy elutes and considered potential nephritogenic autoantibodies. Particularly anti-annexin-A2 antibodies are of great interest. Annexin A2 and anti-Annexin A2 antibodies have been described in kidneys from proliferative LN patients where they co-localize with glomerular IgG and C3 [175,180]. Moreover, Annexin A2 is overexpressed by mesangial cells [175], interacts with β2GP1 and TLR4 leading to proinflammatory and prothrombotic effects, and then promotes autoantibody binding and inflammation [181]. Annexin A2 serves also as ligand for C1q on apoptotic cells, which may contribute to amplify its contribution to SLE pathogenesis [182].

Annexins are a family of 12 proteins able to bind phospholipids in a calcium dependent manner. They play a role in different cellular activities such as trafficking, calcium signaling, cell division, cell growth regulation and apoptosis. Other annexins have been studied in SLE but their association has been questioned due to controversial results [183].

Anti-α-actinin antibodies have been proved as plausible contributors to the pathogenesis of LN [184]. In some lupus-like mouse models, α-actinin immunization generates nephritogenic autoantibodies [185] and anti-α-actinin antibodies are found in serum and kidney elutes of LN models [186]. In humans, SLE patients with active nephritis show an increased levels of anti-α-actinin antibodies in their sera when compared to SLE patients without LN [187]. However, further studies are necessary since longitudinal studies failed to demonstrate their correlation with renal involvement and its utility as a biomarker is questionable since these autoantibodies are found in other autoimmune diseases such as autoimmune hepatitis type 1 (AIH-1) [187,188].

To better explain SLE pathogenesis and due to limited specificity and sensitivity for SLE of the existing autoantibody assays, the use of the last advances in array technology for new autoantigen as disease activity biomarker screening has been encouraged. As a result, Onishi *et al.* described ribosomal RNA-processing protein 8 (RRP8) and spermatid nuclear transition protein 1 (TNP1), both being nuclear proteins, involved in histone modification and chromatin condensation respectively, as novel autoantigens specific for LN [189]. Haddon *et al.* revealed anti-BAFF antibodies to be associated with proliferative nephritis in pediatric SLE but also autoantibodies to collagen IV, collagen X and aggrecan which are extracellular matrix structural proteins [190]. We also reported novel autoantigens by protein array screening as being associated with active nephritis and hypocomplementemia: APEX nuclease 1 (APEX), N-methylpurine-DNA glycosylase (MPG), and CAP-GLY domain containing linker protein family member 4 (CLIP4). These nuclear proteins may be of interest to better understand SLE pathogenesis since some of them are targeted by both IgG and IgE autoantibodies, whereas others seem to be targeted only by autoantibodies of IgE isotype [66].

Some SLE patients develop antibodies against cytokines such as IFNα and BAFF, two crucial molecules demonstrated to be involved in the pathogenesis of the disease. Anti-IFNα antibodies were detected in ~25% of SLE patients and some neutralizing properties have been attributed to them. Indeed, lower serum type I IFN bioactivity, reduced IFN-pathway activity, and lower disease activity were described in these patients. Moreover, the presence of anti-IFNα antibodies was associated with a decreased expression of BAFF, explaining the lower levels of autoantibodies in these patients [191]. On the other hand, the neutralizing effect of anti-BAFF antibodies is not clear since elevated IFNα signature, increased levels of pro-inflammatory cytokines and chemokines, and a higher disease activity was reported in positive patients [192,193]. Still, developments of monoclonal antibodies targeting these cytokines as novel therapeutic strategies for SLE show promising effects in influencing the course of the disease [34,192].

## 4. Autoantibody Isotypes: Classes and/or Subclasses Matter

The different immunoglobulin isotypes (IgM, IgD, IgG, IgA and IgE) are defined by the distinct constant regions in their heavy chains (Cμ, Cδ, Cγ, Cα or Cε, respectively) and diverse tissue distribution to react adequately against different types of pathogens [194]. While IgD is generated through alternative splicing of the primary (germline) transcripts that encode IgM, the other classes are developed through class-switch DNA recombination (CSR) in which a replacement of the Cμ exon with Cγ, Cα or Cε occurs (the antigen-binding variable region remains unaltered). The induction of CSR requires primary and secondary stimuli. The primary induces the expression of AID (activation-induced cytidine deaminase) through T cell-dependent or T cell-independent ways. The secondary stimulus is necessary for directing class switch to IgG, IgA or IgE. The T-cell dependent CSR occurs mainly in germinal centers by the engagement of CD40 on B cells and CD154 on CD4+ T_FH_ cells. CD40 engagement induces CSR strongly towards IgG1 and IgE isotypes in the presence of IL-4 and moderately towards IgG2a and IgA isotypes in the presence of IFN-γ (in mice but not in humans) and TGF-β, respectively. The T-cell independent CSR occurs through the dual engagement of Toll-like receptors (TLR) and B cell receptor for antigen (BCR) (TLR–BCR); of transmembrane activator and CAML interactor (TACI) and BCR; or TLR–TACI. TACI is a TNF receptor family member (TNFRSF13B) specific for B cell-activating factor (BAFF) and a proliferation-inducing ligand (APRIL). Additionally, affinity maturation of an antibody is reached by somatic hypermutations (SHM) which is induced by AID and consists in a high rate of several point mutations in the variable region of the antibody [194]. All of the above cells and factors are known to be involved in SLE and/or autoimmunity. Therefore, autoantibody isotype studies may lead to a better understanding of SLE pathogenesis and to the development of more specific and more sensitive autoantibody arrays able to describe precisely the current clinical state of a given SLE patient.

### 4.1. Natural Antibodies

Natural antibodies are constitutively secreted immunoglobulins detected after birth in neonates or in adults prior to an infection. They are produced by B-1 cells before any immune activation and external antigen exposure [195]. B-1 cell development involves interactions with autoantigens suggesting a positive clonal selection which promotes self-reactivity to non-protein antigens [196]. In mice, B-1 cells are the source of up to 80% of natural IgM antibodies which *V(D)J* genes renders a low rate of somatic hypermutation and therefore show low affinity towards its target displaying either polyreactivity towards a limited range of self-antigens or a great mono-specificity for its binding self-ligand [197]. B-1 cells can enter a follicular pathway and undergo somatic hypermutation and class switching to produce higher-affinity natural IgG or IgA antibodies, mainly under autoimmune conditions such as SLE [198].

The protective role of natural IgM autoantibodies has been studied extensively, revealing their contribution to phagocytosis enhancement, to recognition of apoptotic cells amplification and to pro-inflammatory milieu reduction [199]. Indeed, mice deficient in IgM have defects in apoptotic clearance and have an increased susceptibility to develop a lupus-like disease with the production of IgG autoantibodies to nuclear antigens [200]. Treatments with monoclonal IgM anti-dsDNA antibodies can delay the onset of lupus-like disease in (NZB x NZW) F1 mice [201] and lower the disease in MRL/lpr mice [202]. Moreover, the treatment with a recombinant self-reactive IgM was recently shown to reduce levels of pro-inflammatory T_H_17 cells and autoimmune disease in *FcγRIIB*/*TLR9* double deficient mice [203].

The binding of the natural IgM antibodies to the surface of apoptotic bodies is mediated through the recognition of some “eat-me” signals which includes C1q, MBL, collectins and pentraxins. The high potential valency of the polymeric IgM enhances the recruitment of more MBL and C1q, therefore promoting phagocytosis [197]. Natural IgM antibodies also bind to oxidized lipids such as phosphorylcholine (PC) and the small oxidation-associated determinant (MDA) also detected on apoptotic cells. Anti-PC IgM antibodies are found in healthy individuals. However, in SLE discordant twins the levels of IgM anti-PC is higher in the unaffected twin [204] and higher levels of IgM anti-PC are correlated with lower disease activity and organ damage [205]. These data strongly suggest a beneficial regulatory role of IgM and that their dysregulated production may contribute to SLE pathogenesis. Indeed, self-reactive IgM autoantibodies may have beneficial effects by protecting from uncontrolled inflammation. Thus, high levels of anti-phospholipid IgM antibodies (β2GP1, cardiolipin) in lupus patients have been found to be associated with less frequent renal disease manifestations [206]; anti-dsDNA IgM have been inversely correlated with glomerulonephritis in SLE [64,65] and the diverse profile of autoreactive IgM antibodies detected in lupus patients by microarray studies was correlated with lower disease activity [207].

The knowledge about the immune-regulatory and homeostatic roles of natural IgG and IgA antibodies is still in its early stages, mostly due to the difficulty to separate IgG-spontaneously secreting B cell clones from the stimulated clones. Recently, it has been suggested that antibodies produced in a T cell-independent manner like natural antibodies, exhibit a sialylated pattern, which confers an anti-inflammatory nature compared to antigen-specific antibodies which have agalactosylated and/or asialylated patterns and show pro-inflammatory properties [208].

### 4.2. IgD Antibodies

While a contribution of IgD in inflammatory diseases has been described [209], little is known about their involvement in SLE pathogenesis or autoimmunity in general. The levels of circulating IgD are very low and exhibit a very short half-life attributed to proteolysis. IgD antibodies are mainly expressed on the membrane surface of B cells regulating the B cell fate at specific developmental stages through changes in activation status [210,211]. Guo *et al.* described that the IgD deficiency in the lupus-prone *lpr* mice induces an increased production of autoantibodies (IgG2a, IgG2b and IgM) and a more severe nephritis, whereas the B cell survival and clonal expansion were not affected [212].

### 4.3. IgG Antibodies

The IgG is the most common isotype in peripheral blood, and is divided in different subclasses depending on their Fc region molecular structure. In humans four subclasses are described (IgG1-4), and five are defined in mice IgG1, IgG2a/c, IgG2b and IgG3. The different subclasses have shown distinct pathogenic properties based on the activation abilities of the enzymatic complement cascade as well as distinctive affinity and specificity properties to bind Fcγ receptors (FcγR).

In mice, IgG2a and IgG2b subclasses of autoantibodies were described to exhibit the highest pathogenic activity by inducing anemia after the injection of anti-erythrocyte antibodies derived from the autoimmune-prone NZB mice. Moreover, they bind efficiently FcγRIII and FcγRIV and can activate complement [213]. IgG3 subclass of autoantibody is able to activate complement and has the unique property to form self-associating complexes and induce glomerulonephritis and vasculitis. On the other hand they are not able to bind to any Fcγ receptor [213]. The IgG1 antibody is considered the least pathogenic; it binds FcγRIII but lacks the property of fixing complement through the classical pathway [213]. These findings were supported by the studies performed in MRL-*FAS^lpr^* mice and MRL.*Yaa* mice, in which the progression of the LN was correlated with an enhanced expression of IFN-γ and an increased production of IgG2a and IgG3 [214], lately described to be a T-bet dependent mechanism [215].

The results obtained in mice are less transferable to humans since the IgG subclasses properties are different. Thus, human IgG1-3 subclasses can activate C1q with the following efficacy: IgG3 > IgG1 > IgG2, whereas IgG4 shows minor reactivity to C1q [63]. Nevertheless, IgG2 (resembling more the pathogenicity observed in lupus-like mouse models) was found to be the major isotype detected in renal biopsies of LN patients. Their shown predominant specificities were against α-enolase, annexin AI (podocyte proteins), histone 3 and DNA [176]. The activation of TLR9 by DNA intensively exposed through a NETosis mechanism has been proposed to explain the IgG2 class switching [216] but further studies with regards to their pathogenicity need to be revealed.

IgG1 deposits were also detected at the glomeruli of LN patients [217], and a positive correlation was found between the IgG1 HMC (cultured human mesangial cells) binding and the mesangial immune deposition in LN renal biopsies [62].The restriction of the IgG subclass in autoimmune diseases seems to depend on the antigen targeted [218]. Thus, anti-dsDNA antibodies were considered to be mostly IgG1 and IgG3 (no anti-dsDNA IgG4 detected [42]); anti-SSA/Ro, anti-SSB/La and anti-U1-RNP are IgG1; anti-Sm, anti-ribosomal P are IgG1 and IgG2; anti-cardiolipin are mostly IgG2 [219].

### 4.4. IgE Antibodies

The pathogenic properties attributed to IgE antibodies were broadly studied in the context of allergy and helminth infection and related mainly to their ability to bind to the high-affinity receptor FcεRI on the surface of mast cells and basophils inducing degranulation upon crosslinking by antigens. However, IgE dysregulation has been reported in other inflammatory processes [220] and autoreactive IgEs were found to play a key point in amplifying autoimmunity by an FcεRI-dependent activation of basophils [42]. This would suggest different pathogenic properties of IgE beyond allergy responses. Indeed, we found a high prevalence of autoreactive IgE in SLE against four of the most common autoantigens (dsDNA, Sm, Ro/SSA, La/SSB) and three novel autoantigens (MPG, APEX and CLIP4) [66]. Moreover, when IgE is absent, the disease onset and progression of three lupus-like mouse models (*Lyn*^−/−^, *FcγRIIB^−/−^* and *FcγRIIB*^−/−^ *Yaa*) were described to be delayed [221].

The production of IgE antibodies requires CSR but contrary to IgG, can occur sequentially in germinal centers through the IgG1 intermediate (leading to an extensive process of affinity maturation by SHM) or directly in peripheral tissues producing lower affinity IgEs [222,223]. Although high affinity IgE antibodies are produced in response to an allergen [224], the presence of low-affinity IgE antibodies was implied as few somatic point mutations in the variable region of IgE sequence were reported [225] and the majority of allergen-specific IgEs in the blood of allergic subjects is thought to be locally produced [226]. These differences can be discriminated by the FcεRI itself and elicit distinct molecular mechanisms that are interpreted in different outcomes *in vivo* [14]. Even though the presence of autoreactive IgE in SLE raises many questions, their pathogenicity may be linked to differences in affinity. Thus, recently an increased number of IgE+ plasma cells derived from germinal center B cells after FAS inactivation was reported [227]. These findings are of profound interest considering the high autoimmune predisposition of individuals with FAS/FASL mutations.

### 4.5. IgA Antibodies

IgA antibodies are known to be important players in maintaining systemic and mucosal homeostasis. They protect mucosal surfaces from the invasion of pathogenic microorganisms and promote either powerful pro-inflammatory or anti-inflammatory effects through their interactions with different receptors on immune and non-immune cells [228]. IgA autoantibodies are detected in several autoimmune diseases being IgA nephropathy (IgAN) the most common IgA-associated disease, which is characterized by the deposition of polymeric IgA1 in the kidney [229]. LN is described to have detectable deposits of IgA in the glomeruli, along with IgG, IgM, C3 and C1q. Some SLE patients were reported to have nephritis with mainly IgA deposits, which were considered IgAN patients. However this classification remains controversial since many authors believe this feature to be a clinical subtype of SLE [230].

Despite the contradictory results obtained due to detection methods, anti-dsDNA IgA autoantibodies were described to be elevated in SLE patients showing a positive correlation with disease activity and glomerulonephritis. Moreover, anti-dsDNA IgA might improve the diagnosis of SLE since 7.5% of SLE patients are negative for dsDNA IgG but positive for dsDNA IgA [67].

ANA IgAs were described to be associated with cutaneous manifestations of SLE. Thus, the IgA isotype was differentially elevated in Discoid lupus erythematosus (DLE) [54] (the most common cutaneous chronic manifestation of SLE [231]), and 38% of DLE patients show IgA deposits in the dermal-epidermal junctions [55]. Additionally, in a lupus-like disease model, MRL/*lpr*, which develops cutaneous lesions, the presence of IgA anti-desmoglein 3 was reported to be highly correlated with skin disease aggravation [232].

The presence of anti-β2GP1 IgA was found to be highly prevalent in SLE and mostly associated with thrombotic events [79]. The pathogenic properties were identified when anti-β2GP1 IgA isolated from patients was injected into mice inducing femoral vein clotting [233]. All these scientific evidences might justify the anti-β2GP1 IgA incorporation into the SLICC classification criteria for SLE [49]. However, technical issues in the detection of IgA antibodies, reported in many studies, may affect the interpretation of the results and a verification of the assays is suggested [67,233].

## 5. The 9G4 Idiotype: The Unique Sequence of Autoantibodies

A particular idiotype, designated as 9G4, is characterized by the expression of a single VH gene (VH4-34), is identified in 40% of SLE patients and its levels fluctuate with disease activity. The 9G4 idiotype was detected on a variety of autoantibodies including dsDNA, ssDNA, cold agglutinins and myeloperoxidase (MPO) [234], among others. Although the levels of 9G4 antibodies in healthy controls are low or undetectable, the presence of VH4-34 +B cell repertoire is detected in almost 10% of mature B cells. The study of autoreactive VH4-34 B cells has helped to understand the regulation of B cell tolerance, thus VH4-34 cells are censored at multiple checkpoints during B cell development and are absent from the plasma cell (PC) compartment of healthy individuals but highly expressed in SLE plasma cells [235]. Recently it was described that during SLE flares there is a highly polyclonal repertoire of VH4-34 autoantibodies (which do not recognize the most prevalent lupus autoantigens) from the clonal expansion of circulating antibody-secreting cells. However, a different subset of activated naïve B cells was an important source of antibody-secreting cells and autoantibodies in SLE [48].

## 6. Fc Receptors: The Autoimmune Effector Bridge

The pathogenic potential of autoantibodies is attributable to the combined action of the self-antigen binding properties of the Fab regions as well as the effector functions associated with the Fc region of the different Ig isotypes. The Fc regions are recognized by Fc receptors (FcR) localized on the cellular membranes. Moreover, the biological responses induced by their engagement depend on the cellular and tissue distribution of these FcRs. Once the binding and/or the crosslinking of the Igs with the Fc receptors occurs, different activating or inhibitory effector responses are induced. The resulting pro- or anti-inflammatory cellular responses depend on the Immunoreceptor Tyrosine-based Activation Motif (ITAM) and Immunoreceptor Tyrosine-based Inhibition Motif (ITIM) involved in intracellular signaling cascades [236]. Depending on the valency of the bound isotype, some Fc receptors associated with some ITAM motives are able to induce activating (large aggregation) or inhibitory (low valency) signals (ITAMi) [228].

IgG auto-antibodies are the most studied in SLE isotypes among all the described isotypes and the understanding of FcγRs in the pathogenesis of the disease has given the most extensive advances.

Humans FcγRs are differentiated into nine distinct classes: the classical ones FcγRI, FcγRIIA, FcγRIIB, FcγRIIC, FcγRIIIA and FcγRIIIB; and the non-classical FcRn, FcRL5 and TRIM21. FcRn and TRIM21 are localized intracellularly, binding IgG after its internalization. In mice, there are only 5 different FcγRs described: FcγRI, FcγRIIB, FcγRIII, FcγRIV, and FcRn. Although human and mouse FcγR share the same nomenclature, differences in binding abilities and expression pattern have been described [237].

Allelic variations in the genes encoding the FcγRs have been identified as heritable susceptibility factors for SLE and LN. The study of the functional alterations, one genetic variant may produce, has been the target in the understanding of genetic susceptibility. Thus, many promising results in SLE have been published [13]. The *FCGR2A*-R131 genotype reduces the binding ability of the FcγRIIA to IgG2, decreasing its phagocytosis capabilities and processing of ICs, and inducing the accumulation of IgG2-anti-C1q autoantibodies. The *FCGR3A*-F176 variant shows a reduced binding affinity for IgG1 and IgG3 and segregates preferentially with affected SLE individuals. The FCGR3B*02 allele (NA2) has been associated with SLE susceptibility and to a markedly decreased capabilities of FcγR-mediated phagocytosis of neutrophils reducing clearance of ICs. The *FCGR3B* low-copy number duplication has been correlated with renal involvement in a lupus-like model and in humans [13].

The -169C>T SNP in FCRL3 has been associated with SLE in some ethnic groups [238]. This polymorphism alters the NFκB binding site and is associated with FCRL3 mRNA and surface protein expression [239]. This polymorphism has been associated with the presence of autoantibodies in SLE [240], however, the pathogenic mechanism remains unclear.

There is a constant evidence of a reduced FcγRIIB function in SLE [241]. FcγRIIB is described to be an important negative regulator in B cell signaling and a decreased expression of FcγRIIB on memory B cells and plasmablasts from patients with active SLE compared to quiescent patients has been detected [242]. In the *FCGR2B* gene, some variants show differences in susceptibility among populations. The transmembrane variant *FCGR2B*-T232 has been associated with SLE in Asians showing no variation in Caucasian populations. The promoter *FCGR2B-2.B4* (-386C/-120A) haplotype has been associated with SLE in Americans with European origin. The transmembrane genetic variant showed less inhibitory effects in macrophages on FcγRI- and FcγRIII-mediated phagocytosis, superoxide generation, cytokine production and MHC class I and II expression [243].

The greater findings have been detected when studying mouse models, showing the involvement of FcγRIIB in the autoimmunity pathogenesis and B cell tolerance. Thus, the FcγRIIB-deficient mice on C57Bl/6 background spontaneously develop autoantibodies and an IC-mediated disease [244]; the transgenic restoration of FcγRIIB on B cells in MRL/*lpr* [245] and in (NZBxNZW) F1 [246] mice reduces the development of lupus like phenotype.

## 7. Conclusions

In SLE, a high repertoire of auto-antibodies has been described. Their role in the pathogenesis of the disease may be differentiated not only by the self-antigen exposition they are specific to but by their isotype, by the receptors they bind and even by the affinity to the antigen they recognize. Many of the autoantibodies are not specific for SLE; and can be detected in other inflammatory diseases or in healthy controls. Many tolerance checkpoints are target to promote autoreactivity, helping to positively select the self-reactive-specific B cell clones. Additionally, the abundance of self-antigens induced by the exacerbated apoptosis, NETosis or by the imperfect clearance might explain the wide panel of antibodies specific in SLE. However, it has been shown that the mechanism and/or location of autoantibody-secreting cells development and activation may also influence the profile of antibodies. All these findings show once more the great complexity of the disease and that several pathogenic mechanisms can often lead to the same phenotype. A closest characterization of serological markers such as autoantibodies (isotype and specificity) and their association with SLE organ damage and/or white blood cell activation status represent a big challenge for the biomedical community, but appears to be necessary for the development of better diagnosis, prognosis and even therapy for SLE patients. 
